# Ecology, Floristic–Vegetational Features, and Future Perspectives of Spruce Forests Affected by *Ips typographus*: Insight from the Southern Alps

**DOI:** 10.3390/plants14111681

**Published:** 2025-05-31

**Authors:** Luca Giupponi, Riccardo Panza, Davide Pedrali, Stefano Sala, Annamaria Giorgi

**Affiliations:** 1 Centre of Applied Studies for the Sustainable Management and Protection of Mountain Areas—C.R.C. Ge.S.Di.Mont., University of Milan, Via Morino 8, 25048 Edolo, Italy; riccardo.panza@unimi.it (R.P.); stefano.sala1@unimi.it (S.S.);; 2 Department of Agricultural and Environmental Sciences—Production, Landscape and Agroenergy, University of Milan, Via Celoria 2, 20133 Milan, Italy

**Keywords:** *Picea abies*, snag, species distribution models, bark beetle, plant succession, plant ecology

## Abstract

In recent years, many spruce (*Picea abies* (L.) H. Karst., Pinaceae) forests have been severely affected by bark beetle (*Ips typographus* L., Coleoptera: Curculionidae) outbreaks in the Southern Alps, but their ecological impacts remain poorly studied. We analyzed the distribution, ecological, and floristic–vegetational characteristics of forests recently affected by the bark beetle in the upper basin of the Oglio River (Northern Italy) and developed a MaxEnt model to map forests with a bioclimate more prone to severe insect attacks in the coming decades. The results showed that the spruce forests affected by the bark beetle are located exclusively in the submountain and mountain belts (below 1600 m a.s.l.) and that 85% of them are found in areas with high annual solar radiation (>3500 MJ m^−2^). The predictive model for areas susceptible to severe bark beetle attacks proved highly accurate (AUC = 0.91) and was primarily defined by the mean temperature of the dry winter quarter (contribution: 80.1%), with values between −2.5 and 2.5 °C being particularly suitable for the pest. According to the model, more than 58% of the current spruce forests in the study area will exhibit high susceptibility (probability > 0.7) to severe bark beetle attacks by 2080. The floristic–vegetational and ecological analysis of plant communities of 11 bark beetle-affected areas indicated that more thermophilic and significantly different forest communities (in both floristic and physiognomic terms) are expected to develop compared to those of pre-disturbance. Furthermore, the high coverage of spruce snags/standing dead trees appears to accelerate plant succession, enabling the establishment of mature forest communities in a shorter time frame.

## 1. Introduction

Natural disturbance regimes (e.g., windstorms, fires, insect outbreaks) are intensifying because of climate change, often with negative consequences on the ecosystem services provided by boreal forests [1]. Biodiversity, timber production, tourism, clean water supply, hydrogeological stability, and landscapes are all impacted by bark beetle outbreaks. Although these outbreaks are a natural component of forest dynamics, we are compelled to manage them due to the increasing threat to our economy, health, and cultural heritage [2].

The European spruce bark beetle (*Ips typographus* L., Coleoptera: Scolytinae) is the most significant pest of the Norway spruce (*Picea abies* (L.) H. Karst., Pinaceae), which, in turn, is one of the most important timber tree species in Europe. For centuries, the spruce has been prioritized above other species for its economic value, in the Alps and elsewhere in Europe [3,4]. The spruce has been artificially spread to latitudes and elevations beyond its ecological optimum, where trees face continuous stress and are consequently less able to defend themselves from pests [5]. In spruce forests, *I. typographus* typically persists at low population densities (endemic phase), breeding only in dying or severely weakened trees. However, in the presence of large numbers of stressed trees—such as those affected by windstorms or drought—this bark beetle can undergo population explosions lasting up to 10 years (epidemic phase), during which it also attacks healthy trees [2,6]. In normal conditions, healthy trees repel bark beetles with resin, but this defense is not sufficient during epidemic phases, and it is even weaker in stressed trees. Furthermore, pure spruce stands facilitate *I. typographus* expansion, providing a continuous source of breeding substrate for the beetles [7].

*I. typographus* is highly adaptable, adjusting its voltinism (number of generations per year) according to temperature and photoperiod. It can produce one generation annually at higher latitudes and elevations but up to three generations in milder conditions [5,8]. Rising temperatures and more frequent droughts promote *I. typographus* multivoltinism while simultaneously stressing spruce trees, weakening their natural defenses against this pest [9,10,11]. Consequently, over recent decades, large-scale spruce diebacks have been increasingly observed across Europe [12], transforming a long-standing forestry issue into a matter of public concern, with significant economic, social, and political implications [2]. In the Southern Alps, an unprecedented outbreak of *I. typographus* began in 2015, dramatically exacerbated by the 2018 extreme windstorm “Vaia” and subsequent droughts. Storm Vaia (also known as Storm Adrian) compromised 8.5 million m³ of timber over six alpine regions in Italy, including Lombardy [13,14].

Possibly spreading from Central and Northern Europe, the spruce completely colonized the Alps during the end of the Pleistocene, around 10,000 years ago [15]. Currently, in the Southern Alps, spruce forests are primarily found in the mountain, high-mountain, and subalpine belts, forming diverse plant associations of *Piceion excelsae* phytosociological alliance (*Piceetalia excelsae* order, *Vaccinio-Piceetea* class) [16]. Due to human intervention, driven by the economic value of the Norway spruce, this species is often found in the hilly and submountain belts (or even at lower elevations), forming secondary vegetation that replaces broadleaf forests such as beech (*Fagus sylvatica* L., Fagaceae) or sessile oak (*Quercus petraea* (Matt.) Liebl., Fagaceae) woods. Despite its ecological plasticity [17], the climatic optimum of the Norway spruce in the Southern Alps is found in the high-mountain and subalpine belts, primarily because this species is of the Eurosiberian chorological type [18], adapted to long, cold winters and short, warm summers.

Several biotic and abiotic factors are closely linked to the spruce and *I. typographus* dynamics, including breeding substrate availability, natural enemies (predators and parasitoids), forest composition (mixed or pure stands), intraspecific competition, weather (temperature and rainfall), and solar radiation [19,20,21]. Faccoli and Bernardelli (2014) [7] indicate that forest composition and elevation are major drivers of *I. typographus* dynamics in the Southern Alps. Indeed, mixed forests (composed of multiple tree species) and those at higher elevations are less susceptible to bark beetle damage compared to pure spruce stands at lower elevations. These patterns align with observations from Central and Northern Europe [19,22].

Given the rapid pace at which the bark beetle is destroying the Norway spruce forests of the Southern Alps (and the resulting ecological, economic, and hydrogeological problems), there is growing interest among researchers in understanding how *I. typographus* outbreaks will respond to the fast pace of climate change in mountainous regions [4]. However, in the Southern Alps and Europe in general, the processes of vegetation succession following bark beetle outbreaks remain poorly explored/understood. Researchers have primarily focused on forestry implications and tree regeneration [23,24], largely leaving the floristic and ecological aspects of plant communities (pre- and post-bark beetle attack) unexplored [25]. This gap should be addressed, if for no other reason than that vegetation can be used as a “super-indicator”, useful for understanding the mechanisms that regulate forest ecosystems (and others) and thus supporting the proper management of these ecosystems.

This research aims to provide further information regarding the environmental characteristics of the spruce forests in the upper basin of the Oglio River (Southern Alps) recently affected by severe bark beetle infestations by analyzing their geographical, bioclimatic, and vegetational features. Furthermore, through the application of species distribution models and the interpretation of floristic–vegetational data, it seeks to provide an overview of the forests in the study area that are highly likely to be infested/destroyed by the bark beetle in the coming decades and how vegetation succession may proceed in order to provide tools for improving the resilience of these forest ecosystems.

## 2. Materials and Methods

### 2.1. Study Area and Sampling Sites

**Figure 1:**
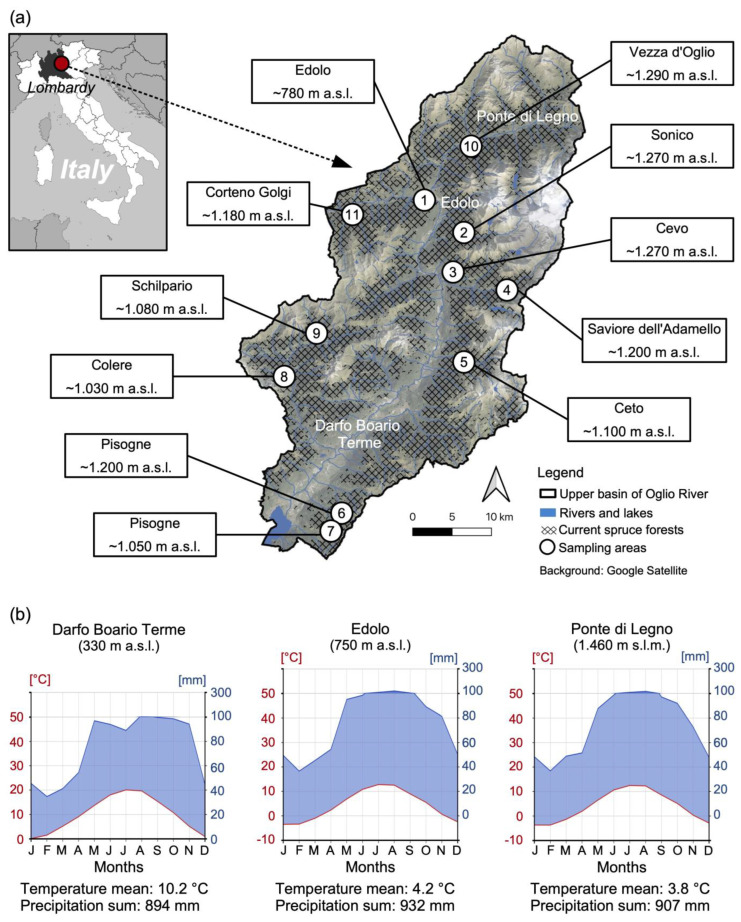
Map of the study area (UBOR) with sampling sites (the identification code for each sampling site is indicated within circles) (**a**), and Walter–Lieth charts developed using Zepner et al. (2020) [30] (**b**).

The upper basin of the Oglio River (UBOR) is in the Southern Alps within the Lombardy region of Italy (Latitude: 46°00′ N, Longitude: 10°20′ E) (Figure 1). It covers a large area of 1444 km^2^ and includes 49 municipalities spread across the Valle Camonica in the province of Brescia and Val di Scalve in the province of Bergamo.

From an orographic perspective, the UBOR is situated between the Central and Eastern Lombard Prealps and the Southern Rhaetian Alps [26]. The Central and Eastern Lombard Prealps are primarily composed of sedimentary limestone rocks, while the Southern Rhaetian Alps feature sedimentary, metamorphic, and intrusive rocks with neutral and acidic reaction [27,28]. The elevation range within the study area is considerable, spanning 3300 m: the southern valley areas (near Lake Iseo) are situated at around 200 m a.s.l., while the highest peak (Mount Adamello) exceeds 3500 m a.s.l.

The climate in the UBOR is quite diverse: in the southern mountainous areas, there is a sub-oceanic climate (with rainfall concentrated around the equinoxes), while in the northern areas, there is a sub-continental climate (with precipitation peaks in summer) [29]. Across the entire UBOR, the driest period occurs during the cold winter months, from December to March (Figure 1).

The vascular flora (tracheophytes) of the UBOR includes 2732 taxa (species and subspecies) [27,31]. The vegetation is largely composed of forests, with types that vary mainly according to altitude, substrate, and land management practices [17,32].

Spruce forests cover about 30% of the forested area in the study region and are primarily located within the mountain and high-mountain vegetational belts, ranging from 700 to 1800 m a.s.l. (https://www.geoportale.regione.lombardia.it, accessed on 1 April 2025). These forests mainly consist of plant communities dominated by spruce, with larch (*Larix decidua* Mill., Pinaceae) and Swiss pine (*Pinus cembra* L., Pinaceae) at higher elevations and more thermophilic trees (such as silver fir, beech, and chestnut) at lower elevations. From a phytosociological perspective, the spruce forests of the mountain and high-mountain belts belong to the *Calamagrostio arundinaceae-Piceetum* association (*Vaccinio-Abietenion* sub-alliance, *Piceion excelsae* alliance, *Piceetalia excelsae* order, *Vaccinio-Piceetea* class), which is the most widespread spruce forest association in this part of Lombardy [32,33]. The *Calamagrostio arundinaceae-Piceetum* association is characterized by abundant moss cover and the presence of tracheophytes, including *Picea abies* (dominant tree), *Larix decidua*, *Betula pendula* Roth (Betulaceae), *Sorbus aucuparia* L. (Rosaceae), *Lonicera nigra* L. (Caprifoliaceae), *Calamagrostis arundinacea* (L.) Roth (Poaceae), *Vaccinium myrtillus* L. (Ericaceae), *Oxalis acetosella* L. (Oxalidaceae), *Luzula nivea* (Nathh.) DC. (Juncaceae), *Saxifraga cuneifolia* L. (Saxifragaceae), and *Phegopteris connectilis* (Michx.) Watt (Thelypteridaceae). At higher elevations, in the subalpine belt, forest communities contain less spruce and more larch, with *Rhododendron ferrugineum* L. (Ericaceae) and *Luzula nivea* in the understory (*Luzulo niveae-Piceetum rhododendretosum ferruginei*) [33]. In the highest subalpine areas, spruce becomes sporadic in larch-dominated forests (*Astrantio minoris-Laricetum deciduae*), where *Pinus cembra* is also present [33,34].

For the study of the vegetation of the spruce forests affected by the bark beetle (and control/unaffected forests), 11 sampling sites were identified across the entire study area, at altitudes ranging from 700 to 1300 m a.s.l. (Figure 1). These sites were selected based on the presence of at least one hectare of forest with over 90% standing dead spruce trees (some of them snapped or with little branches left, hence, for brevity, we will refer to all of them as “snags”), following a bark beetle infestation that occurred in the previous 3–7 years. The withering of these spruces was attributed to bark beetle activity by the regional forest service, which has monitored the spread of the pest over the past decade through satellite imagery and field surveys.

### 2.2. Analysis of the Current and Future Distribution of Forests Affected by Bark Beetle

The analysis of the current forests affected by bark beetles in the UBOR utilized cartographic data (polygon shapefile) sourced from the Geoportal of the Lombardy Region (https://www.geoportale.regione.lombardia.it, accessed on 1 April 2025). The shapefile of bark beetle-affected areas in Lombardy (updated to 2021 with 760 polygons corresponding to approximately 2070 ha of forests affected by bark beetles) was downloaded from the geoportal and analyzed using ArcGis Pro software. Specifically, 241 polygons within the UBOR were considered. For each 50 m altitude interval (from 300 to 2150 m above sea level), the affected area of spruce forest impacted by bark beetle was calculated, as well as the percentage of affected forest relative to the total area of spruce forests (including both pure spruce forests and mixed forests with spruce). For each polygon, the average slope, elevation, and aspect were calculated. These data, along with the geographic coordinates of each polygon, were then used to calculate the theoretical annual global radiation for each area using software developed by ENEA (http://www.solaritaly.enea.it/indexEn.php, accessed on 1 April 2025) to assess whether a relationship exists between bark beetle-affected areas and incident solar energy. The ENEA software computes the theoretical annual solar radiation of an area considering the geographic position, the zenith angle, the atmosphere absorption and diffusion, and the average cloud cover derived from historical data and satellite observations.

Species distribution models (SDMs) were adopted to predict the spatial distribution (current and future) of *I. typographus* in UBOR based on bioclimatic data. Nineteen bioclimatic variables (Table 1) and 14 georeferenced points (occurrence points) where the bark beetle destroyed more than 20 ha of spruce forest in the Lombardy Alps were considered as severely infested areas. The coordinates of the occurrence points were extracted from the Geoportal of the Lombardy Region shapefile, considering the areas where the bark beetle has been particularly destructive. The bioclimatic layers were obtained from the WorldClim 2.1 data website (http://worldclim.org, accessed on 1 April 2025) at a spatial resolution of 0.5 arc-minutes (~0.60 km^2^). All the bioclimatic variables were used to establish the distribution model of *I. typographus* (with high destructive capacity) in UBOR under current climatic conditions (2016–2020) and for three future periods: 2021–2040, 2041–2060, and 2061–2080.

The Shared Socio-economic Pathway (SSP) 2–4.5 future scenario (“Middle of the road scenario”) was considered in this analysis because it represents a plausible future pathway characterized by medium challenges to mitigation and adaptation efforts [35]. The CNRM-CM6-1 global climate model [36], used for evaluating and forecasting the impact of the SSP scenarios on future climate, was acquired from the WorldClim data website. This model, representing the latest fully coupled atmosphere–ocean general circulation model of the sixth generation, was utilized to project the effects of the SSP2-4.5 scenario onto the climate of the periods 2021–2040, 2041–2060, and 2061–2080.

The distribution model of *I. typographus* was generated with MaxEnt [37] using the “dismo” package [38] of R software. MaxEnt (Maximum Entropy) is an algorithm widely used for making predictions of species distribution, particularly well-suited for applications involving presence-only data (occurrence data). MaxEnt estimates the probability distribution of maximum entropy, constrained by the environmental characteristics of known presence locations, to identify areas of suitable habitat. Among its outputs, MaxEnt provides the percentage contribution and permutation importance of each predictor variable. The percentage contribution reflects the influence of each variable during model training, while permutation importance assesses the reduction in model performance when the variable’s values are randomly permuted, thus indicating its independent predictive power. MaxEnt also generates response curves, which illustrate how the predicted suitability for the species changes along the gradient of each environmental variable, helping to interpret ecological relationships.

The accuracy of the model was evaluated by computing the Area Under the Curve (AUC) of the Receiver Operating characteristic Curve (ROC), a widely used and robust approach of model evaluation. The AUC values range from 0 to 1, and the higher the value of AUC, the better the performance of the model.

The final output of the distribution model is a map indicating areas with a high/low probability (0 for low probability; 1 for high probability) of the bark beetle having particularly destructive effects. In this research, the probability of occurrence was transformed into a binary score (presence or absence) considering the threshold of 0.70, and then four presence/absence maps of *I. typographus* in UBOR were created: one considering the current climatic conditions (2016–2020) and three considering the future periods 2021–2040, 2041–2060, and 2061–2080.

### 2.3. Vegetation Data Collection and Analysis

For each sampling site (Figure 1), 6 phytosociological relevés were carried out on 100 m^2^ (10 × 10 m) using the methods of Braun-Blanquet (1964) [39]: 3 relevés were conducted in areas affected by the bark beetle (i.e., those with more than 90% of spruce snags), and the other 3 were conducted in adjacent areas with no spruce snags (control). Given the difficulty in diagnosing whether live spruce trees were affected by the bark beetle (in the early stages of infestation, spruce trees show no symptoms but only little bark holes, which may be located several meters up the trunk), this study conventionally considered “affected forests” to be those with high coverage of standing dead spruce trees (snags), and “unaffected forests” to be those without snags.

For each relevé, tracheophytes of the plant communities were identified using the “Flora d’Italia” dichotomous keys of Pignatti (2017) [18], and their coverage was estimated using the conventional abundance/dominance scale of Braun-Blanquet (1964) [39]: r, rare species in the relevé; +, coverage < 1%; 1, coverage 1–5%; 2, coverage > 5–25%; 3, coverage > 25–50%; 4, coverage > 50–75%; and 5, coverage > 75–100%. In each relevé, two coverage values were assigned for *Picea abies*: one for snag trees and one for live trees. Additionally, the total moss coverage percentage was estimated. The relevés were performed in June-July 2023 and 2024.

The data of the relevés were arranged in a matrix (relevés × species) where Braun-Blanquet abundance/dominance indexes were converted into percentage of plant coverage, as proposed by Canullo et al. (2012) [40] (r, 0.01%; +, 0.5%; 1, 3.0%; 2, 15.0%; 3, 37.5%; 4, 62.5%; 5, 87.5%); subsequently, a power transformation with the exponent 0.5 on these values was carried out according to [41].

Cluster analysis and Detrended Correspondence Analysis (DCA) were performed to identify floristic–physiognomic similarities/differences among the relevés using the “vegan” package of R [42]. Cluster analysis was performed using the Unweighted Pair Group Method with Arithmetic mean method (UPGMA) and the chord distance coefficient [43]. Furthermore, Pearson’s phi coefficient (*Φ*) was used to identify the diagnostic species (plants significantly associated with the different types of vegetation) as proposed by Chytry et al. (2002) [44] and Tichý and Chytry (2006) [45]. The coefficient *Φ* was calculated using the following formula:

(1) $$\Phi =\frac{N\cdot \;n_{p}-n\cdot N_{p}}{\sqrt{n\cdot N_{p}\cdot (N-n)\cdot (N-N_{p})}}$$

where *N* is the number of relevés in the data set, *N_p_* is the number of the relevés in a target group of relevés, *n* is the number of occurrences of the species in the data set, and *n_p_* is the number of occurrences of the species in the target group of relevés. The coefficient *Φ* can assume values from 1 (the species is concentrated in the target relevés group) to −1 (the species is under-represented in the target relevés group). The identification of the diagnostic species was carried out using the “indicspecies” R package [46].

Phytosociological analysis of vegetation was performed using the hierarchical floristic classification system of vascular plant, bryophyte, lichen, and algal communities of the European vegetation [16] and “Prodromo della vegetazione d’Italia” [47]. Therefore, the phytosociological classes of the species of the relevés were assigned (considering only the vegetation classes of the Southern Alps) to determine the ecological features of the plant communities. Additionally, the indices of Landolt et al. (2010) [48] were applied for synecological analysis. Specifically, for each relevé (and groups of relevés), the mean values of T—temperature, K—continentality, L—light intensity, F—soil moisture, R—substrate reaction, N—nutrients, H—humus, and D—soil aeration were calculated. Ecological differences between bark beetle-affected and unaffected forests were determined using Student’s *t*-test. A *p*-value of less than 0.05 was considered statistically significant.

The scientific names of the plant species are in accordance with Pignatti (2017) [18], while the names and the codes of syntaxa follow Mucina et al. (2016) [16]. The statistical analyses were performed using R 3.6.1 software [49].

## 3. Results

### 3.1. Current and Future Distribution of Affected and Susceptible Spruce Forests

**Figure 2:**
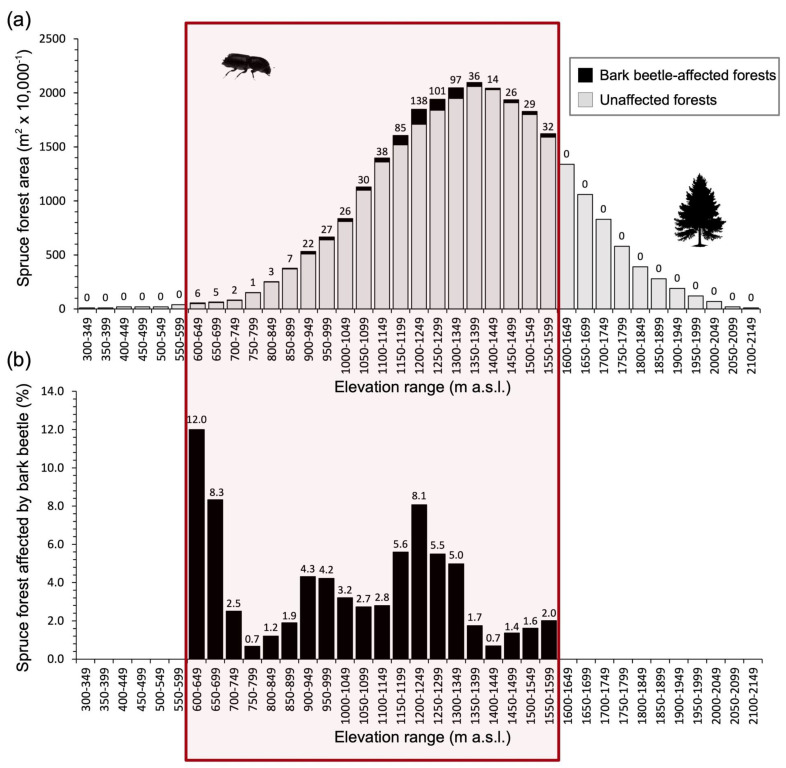
Extent of spruce forests (affected and unaffected by bark beetle) across the different altitudinal ranges of the study area (**a**) and percentage of spruce forest affected by bark beetle (**b**). The numbers above the columns refer to affected forests. The red box highlights the current altitudinal distribution of bark beetle damage.

Based on data from the Geoportal of the Lombardy Region, the study area contains spruce forests (26,800 ha) ranging in altitude from 300 m to 2150 m (Figure 2a). Of these, 88% are in the mountain and high-mountain belt between 800 m and 1700 m a.s.l. The area of forest affected by the bark beetle amounts to 725 ha, equivalent to 2.7% of the total spruce forest area in the study. These affected forests are confined to altitudes between 600 m and 1600 m a.s.l.

The highest rates of spruce forest damage (>8.2%) occur at lower elevations (600–700 m a.s.l.) (Figure 2b). Significant damage is also observed in the elevation band between 1150 m and 1300 m a.s.l., where the percentage of affected forest exceeds 5%. In the upper high-mountain belt above 1600 m a.s.l. and in the subalpine zone, no spruce forests currently show signs of bark beetle infestation.

**Figure 3:**
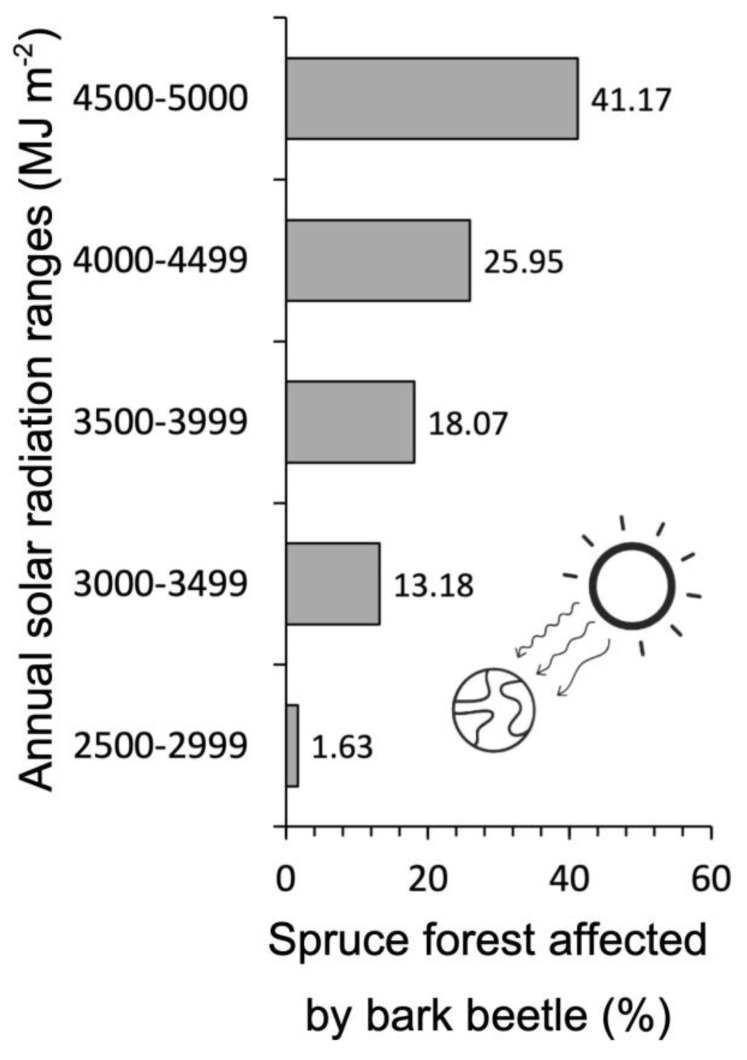
Percentage of spruce forests attacked by bark beetle for each potential annual solar radiation range in the UBOR.

In the study area, the extent of spruce forest affected by bark beetle gradually increases with higher levels of annual solar radiation. In fact, 85% of the infested spruce stands are in areas where the theoretical annual solar energy exceeds 3500 MJ m^−2^ (Figure 3).

**Figure 4:**
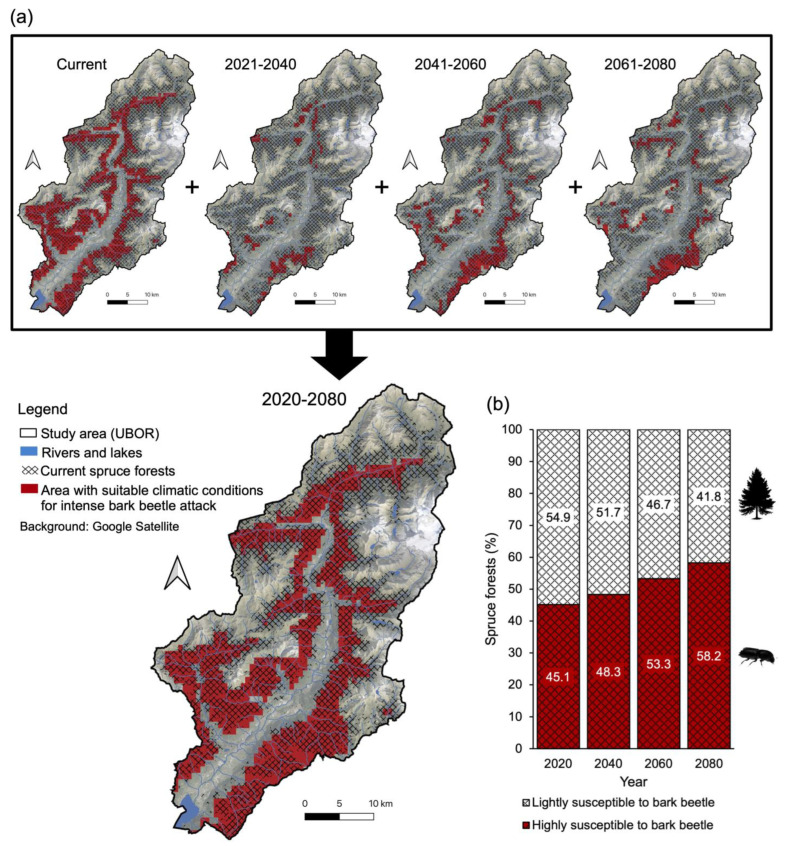
Current and future areas of UBOR with particularly suitable climatic conditions for intense bark beetle attacks (probability of infestation > 70%) returned by the MaxEnt model (**a**), and total percentage of spruce forests with high susceptibility to *I. typographus* from 2020 to 2080 (**b**).

Areas with bioclimatic conditions suitable for the bark beetle to cause significant damage to spruce forests and the percentages of forests lightly/highly susceptible to mass infestations are expected to increase according to our model (Figure 4). The model reveals that areas with suitable climatic conditions for the bark beetle shift in extent and elevation over time (from the current situation to projections for 2080). As a result, the percentage of forests highly susceptible to mass bark beetle infestations is expected to increase from 45.1% to 58.2% over the next 60 years (Figure 4b). Based on habitat suitability maps (Figure 4a), in the future, spruce forests may find optimal conditions (low likelihood of severe bark beetle attacks) above 1800 m a.s.l. in southern areas of the UBOR (lower Camonica Valley) and above 1500 m a.s.l. in northern areas (upper Camonica Valley).

The distribution model generated by MaxEnt, used to produce habitat suitability maps, has high predictive accuracy (AUC = 0.91). Among the 19 bioclimatic variables considered, BIO9 (mean temperature of the driest quarter) contributes the most to the model’s formulation (percent contribution: 80.1%; permutation importance: 77.6%) (Table S1).

**Figure 5:**
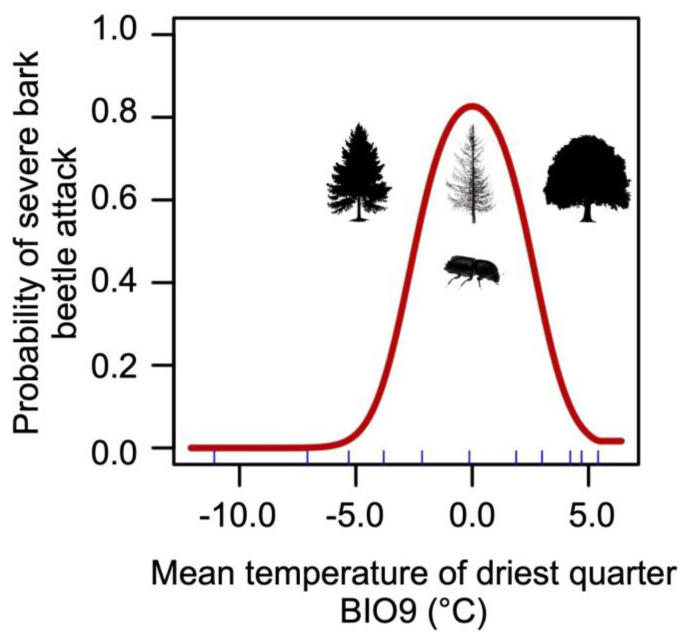
MaxEnt response curve of severe bark beetle attack to the mean temperature of driest quarter variable (BIO9).

The most suitable habitat for severe bark beetle outbreaks occurs where the mean temperature of the driest quarter (which in the study area corresponds to winter) ranges between 4 °C and −4 °C, as showed by the relationship (response curve) between the probability of massive bark beetle infestations and the BIO9 variable (Figure 5). Areas with BIO9 values below −4 °C, typically at higher elevations, are less suitable for the bark beetle (probability of presence < 0.2). Similarly, areas with BIO9 values above 4 °C are also unsuitable for massive bark beetle attack.

### 3.2. Vegetation Features

**Figure 6:**
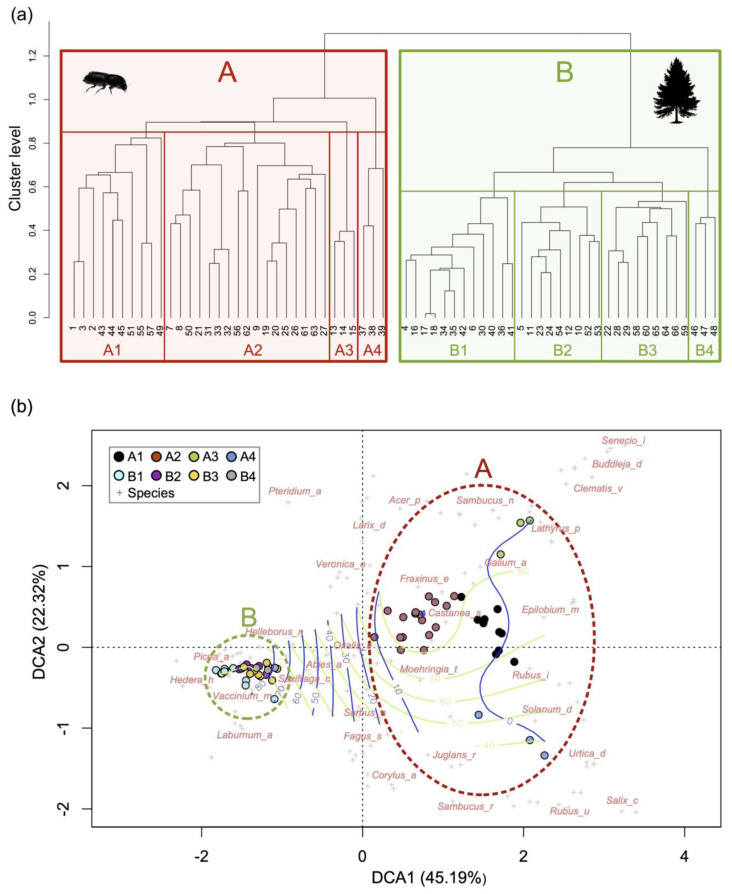
Dendrogram (**a**) and DCA biplot of relevés (**b**). The two main clusters (A—spruce forests affected by bark beetle; B—forests unaffected by bark beetle) and subclusters are highlighted. The DCA biplot is overlaid with blue contour lines indicating the percentage of live spruce cover (blue line) and spruce snag cover (green line). In the DCA biplot, species are marked with crosses, with some species highlighted: *Picea_a*, *Picea abies* (alive); *Hedera_h*, *Hedera helix* Screb. (Ranunculaceae); *Vaccinium_m*, *Vaccinium myrtillus*; *Laburnum_a*, *Laburnum alpinum* (Mill.) Bercht. & J. Presl (Fabaceae); *Helleborus_n*, *Helleborus niger*; *Pteridium_a*, *Pteridium aquilinum* L. (Dennstaedtiaceae); *Abies_a*, *Abies alba*; *Oxalis_a*, *Oxalis acetosella*; *Veronica_o*, *Veronica officinalis* L. (Plantaginaceae); *Larix_d*, *Larix decidua*; *Sorbus_a*, *Sorbus aucuparia*; *Fagus_s*, *Fagus sylvatica*; *Corylus_a*, *Corylus avellana*; *Acer_p*, *Acer pseudoplatanus*; *Fraxinus_e*, *Fraxinus excelsior* L. (Oleaceae); *Moehringia_t*, *Moehringia trinervia* (L.) Clairv. (Caryophyllaceae); *Juglans_r*, *Juglans regia* L. (Juglandaceae); *Castanea_s*, *Castanea sativa*; *Galium_a*, *Galium aparine* L. (Rubaceae); *Sambucus_r*, *Sambucus racemosa* L. (Viburnaceae); *Sambucus_n*, *Sambucus nigra* L. (Viburnaceae)*; Saxifraga_c*, *Saxifraga cuneifolia; Rubus_i*, *Rubus idaeus*; *Epilobium_m*, *Epilobium montanum* L. (Onogranaceae); *Lathyrus_p*; *Lathyrus pratensis* L. (Fabaceae); *Solanum_d*, *Solanum dulcamara* L. (Solanaceae); *Rubus_u*, *Rubus ulmifolius* Schott (Rosaceae); *Urtica_d*, *Urtica dioica* L. (Urticaceae); *Salix_c*, *Salix caprea*; *Clematis_v*, *Clematis vitalba* L. (Ranunculaceae); *Senecio_i*, *Senecio inaequidens*; *Buddleja_d*, *Buddleja davidii*.

A total of 198 species of herbs, shrubs, and trees were identified (Table S2). Figure 6a shows the dendrogram generated by cluster analysis, highlighting two main vegetation types: cluster A and cluster B.

Cluster A includes all relevés conducted in forests affected by the bark beetle, while cluster B comprises those conducted in forests unaffected by the pest. These two clusters are characterized by distinct plant communities (Figure 6b) and are mainly differentiated by the presence of live/dead spruces and diagnostic species listed in Table 2. The diagnostic species of cluster B (*Picea abies*, *Vaccinium myrtillus* and *Saxifraga cuneifolia* L., Saxifragaceae) are characteristic of holarctic coniferous forests (*Vaccinio-Piceetea* phytosociological class), with an understory featuring substantial moss coverage. In contrast, the diagnostic species of cluster A are primarily associated with shrublands of the *Robinietea* class (seral forest-clearing and anthropogenic successional scrub and thickets) and forest edges or clearings of the *Epilobietea angustifolii* class (tall-herb vegetation of forest edges and clearings), as well as spruce snags.

Each main vegetation cluster is further subdivided into subclusters (Figure 6), which reveal floristic/physiognomic differences in plant communities. These differences are mainly attributable to the environmental conditions of the specific survey sites, such as soil type, microclimate, and anthropogenic management or disturbance. For cluster A, four subclusters/plant communities have been identified:

- A1—Plant community dominated by heliophilous shrubs such as *Rubus idaeus* L. (Rosaceae) (the dominant species), typical of clearings, disturbed forests, or areas affected by treefall or logging (*Rubetum idaei* association; *Robinietea* class). In this study, it occurs in areas where the density or coverage of dead spruce trees is low.
- A2—Plant community characterized by a high density and coverage of dead spruce trees, with an understory featuring limited *Rubus idaeus* and abundant *Vaccinium myrtillus*, *Luzula nivea*, ferns, and mosses. It also includes young broadleaf trees (*Fagus sylvatica*, *Sorbus aucuparia*, *Castanea sativa*) typical of mature forest communities with occasional silver fir (*Abies alba* Mill., Pinaceae) and spruce.
- A3—Shrubland with *Rubus idaeus* and a high density of exotic species such as *Buddleja davidii* Franch. (Scrophulariaceae), *Senecio inaequidens* DC. (Asteraceae) and *Erigeron canadensis* L. (Asteraceae). These species are associated with anthropogenic disturbance in areas near roads or settlements where spruce trees have been cut and removed.
- A4—Shrubland composed of thermophilic species, including *Rubus ulmifolius* (the dominant species), *Corylus avellana* L. (Betulaceae), *Populus tremula* L. (Salicaceae), *Fagus sylvatica,* and *Salix caprea* L. (Salicaceae) (Figure 6, Table S2).
- Cluster B can also be divided into four subclusters, although these are less dissimilar compared to those in Cluster A (Figure 6):
- B1—Dense, thermophilic spruce forest with low coverage/presence of herbaceous plants and bryophytes, and with young *Castanea sativa* trees;
- B2—Spruce forest with bryophytes, *Vaccinium myrtillus*, and young broadleaf trees (*Fagus sylvatica*, *Betula pendula*, *Castanea sativa*, *Quercus petraea*, *Acer pseudoplatanus* L., Sapindaceae);
- B3—Spruce forests with well-established spruce regeneration and high coverage of *Oxalis acetosella* and bryophytes in the understory;
- B4—Spruce forests on neutral to basic soils, characterized by basiphilous herbaceous plants (*Sesleria caerulea* (L.) Ard., Poaceae; *Helleborus niger* L., Ranunculaceae; *Carex alba* Scop., Cyperaceae; *Hepatica nobilis* Schreb., Ranunculaceae) and *Fagus sylvatica* (Figure 6, Table S2).

**Figure 7:**
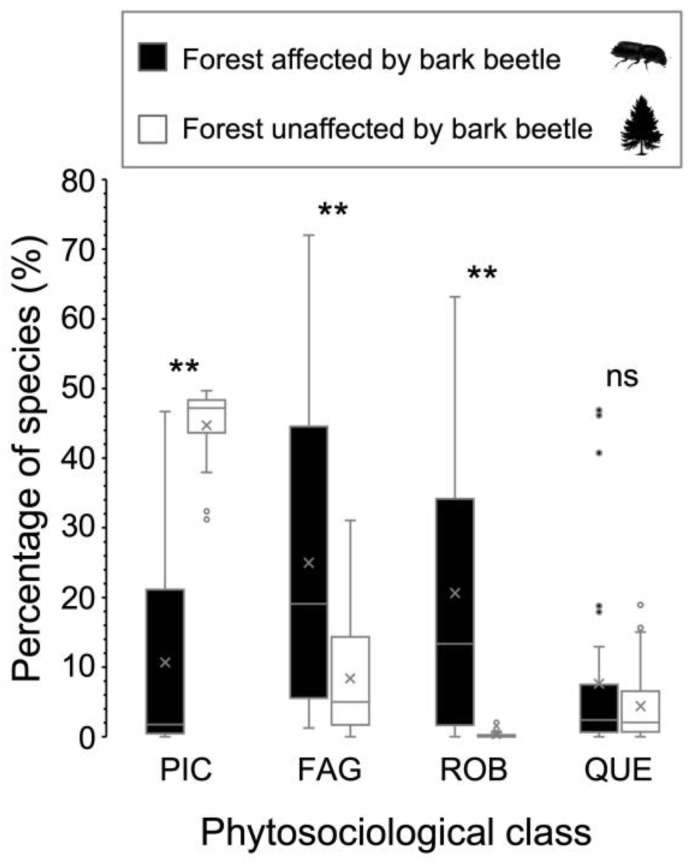
Boxplots of the main phytosociological classes of species identified in forests affected by bark beetles and in those unaffected, including average values (indicated with an “x”). Relevés from affected and unaffected forests have significantly different proportions of species from of PIC, FAG, and ROB classes. The symbols above the boxes indicate significant differences as determined by the *t*-test: **, *p* < 0.01; ns, not significant. Key: PIC, *Vaccinio-Piceetea*; FAG, *Carpino-Fagetea sylvaticae*; ROB, *Robinietea*; QUE, *Quercetea robori-petraeae*.

In general, the forest communities affected by the spruce bark beetle (cluster A) are predominantly composed of species of *Carpino-Fagetea sylvaticae* class (mesic deciduous and mixed forests of temperate Europe) and *Robinietea* class, with a smaller proportion of species of *Vaccinio-Piceetea* and *Quercetea robori-petraeae* (acidophilous oak and oak-birch forests) (Figure 7). Conversely, in spruce forests unaffected by the bark beetle, the percentage of *Vaccinio-Piceetea* species is the highest, followed by those of *Carpino-Fagetea sylvaticae* and *Quercetea robori-petraeae* classes. In cluster B, *Robinietea* species are almost absent (Figure 7).

**Figure 8:**
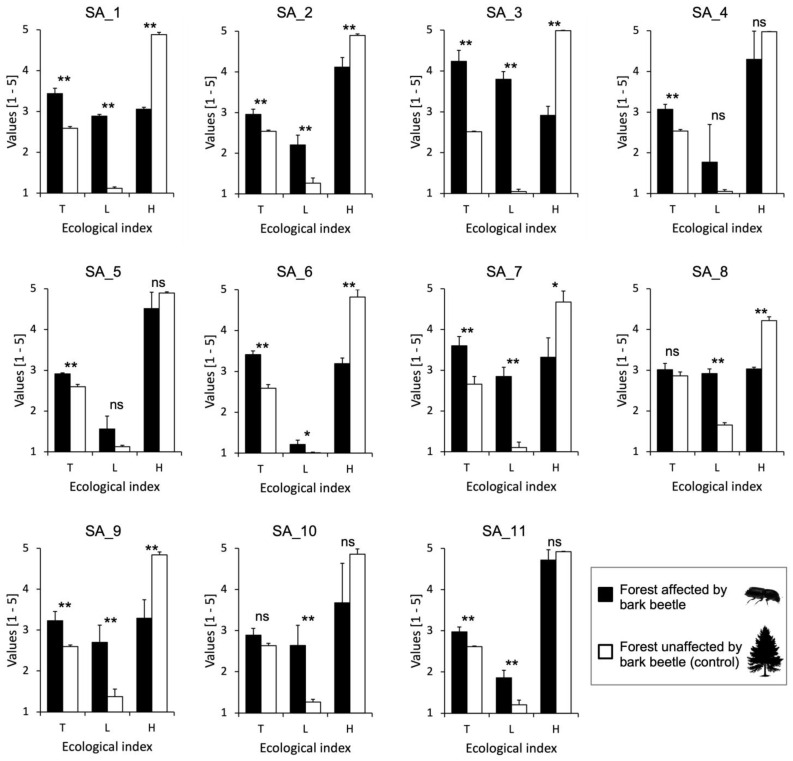
Values of key ecological variables (*p* < 0.05 in more than 60% of the sampling sites) that distinguished the vegetation communities of beetle-affected and unaffected forests across the study sites. Average values of the ecological indices T (temperature), L (light), and H (humus) for plant communities of forests affected and unaffected by bark beetles for each sampling area (SA). The numerical codes for the SAs are the same as those used in Figure 1. The symbols above the bars indicate significant differences in the ecological features of plant communities as determined by the *t*-test: *, *p* < 0.05; **, *p* < 0.01; ns, not significant.

Following the calculation of the ecological indices of Landolt (Table S3), at all sampling sites, communities established after a bark beetle attack were more thermophilic compared to those in unaffected forests. Additionally, the communities in beetle-affected forests consisted of more light-demanding (heliophilous) species that require less organic matter (or litter) in the soil (Figure 8).

## 4. Discussion

The results of the analyses regarding the characteristics of the areas currently affected by the spruce bark beetle provided valuable insights into the ecology of spruce forests and bark beetle outbreaks in the Southern Alps, considering both current climatic conditions and future scenarios. It was evident that in the study area, elevation, solar radiation, and the temperature of the driest quarter of the year are the most relevant variables for the development of severe bark beetle outbreaks. In particular, spruce forests located in areas warmer during the winter months (generally at lower elevations and/or in areas with good exposure to sunlight) were found to be the most susceptible to intense bark beetle outbreaks. These conditions are currently observed below 1600 m a.s.l. in the submountain and mountain vegetation belts, where spruce trees are likely to experience greater stress due to high temperatures, which, in turn, allow the bark beetle to increase the number of generations per year. These results are consistent with Kozhoridze et al. (2024) [19], who found a negative relationship between infestation and elevation, with higher infestation rates below 900 m a.s.l. Also, Jakoby et al. (2019) [50] observed that, in the Swiss Alps, the number of *I. typographus* generations per year is 2 at 500 m of elevation, 1.5 at 1000 m, and 1 at 1700 m. Although there are no studies on the annual number of bark beetle generations in the UBOR, it is reasonable to assume that, at the same elevations as the Swiss Alps, the number of *I. typographus* generations is similar, if not higher, given that the Swiss Alps are located further north (in the inner Alps), where temperatures are (and will be) generally lower compared to those in the Southern Alps [51].

In addition to that, solar radiation—which primarily depends on slope and aspect—also increases the flight activity and voltinism of *I. typographus* [50,52]. This confirms the findings of this research, which revealed that the affected forest area also increases in line with annual theoretical radiation (Figure 3). Monitoring solar radiation, combined with temperature data for a specific area, can enable a precise assessment of *I. typographus* development within a spruce forest. In fact, Baier et al. (2007) [53] developed a model (PHENIPS) based on topoclimatic data for the spatial and temporal simulation of the seasonal development of *I. typographus* at Kalkalpen National Park (Austria), which yielded good results. This model could also be applied in the UBOR region to monitor the expansion of the bark beetle over time, assess the accuracy of the distribution maps produced by MaxEnt (Figure 4), and determine where and when to implement bark beetle containment measures.

In the literature, summer temperature appears to be the most relevant factor in the outbreak dynamics of *I. typographus* [52,54,55]. However, this research suggests that the mean temperature of the driest winter months (BIO9) could be equally important and should be considered when understanding and predicting the distribution (current and future) of bark beetle infestations, at least in the Southern Alps. The results of this study indicate that a high probability (>0.5) of intense bark beetle outbreaks occurs where BIO9 values range between −2.5 and 2.5 °C. This is explained by the fact that winter temperatures within this range result in higher survival rates for overwintering individuals (particularly eggs, larvae, and pupae), leading to more severe infestations in spring and summer [56]. Areas with BIO9 values that are too high (>5 °C) are entirely unsuitable for the bark beetle (Figure 5), primarily because these warmer zones lack spruce forests and are instead dominated by broadleaf forests, which are not susceptible to *I. typographus*. Similarly, areas with excessively low winter temperatures (BIO9 < −5 °C), typically located at higher elevations (subalpine and alpine belts), are also unsuitable for the bark beetle. This is due to several factors, including extreme minimum temperature peaks reducing the survival rate of overwintering individuals, the absence of spruce forests at the highest elevations in the study area (the alpine belt in the UBOR is dominated by grasslands, rocks, scree, and glaciers), and/or the presence of mixed forests of spruce, larch, and Swiss pine in the subalpine belt. In fact, in forests composed of various tree species (mixed forests), spruce trees are less susceptible to bark beetle attacks because they are harder for the pest to locate due to the lower presence/concentration of host volatiles emitted by spruce wood [57]. Additionally, within the context of plant–insect interactions, it is well known that volatile compounds emitted by non-host plant species can interfere with the insect’s response to aggregation pheromones [58]. Such knowledge supports the strategies of converting pure spruce forests into mixed forests, which can significantly reduce bark beetle damage [59].

To date, no damage caused by *I. typographus* has been recorded above 1600 m a.s.l. in the study area, and there is a low percentage (<2%) of damaged spruce forests in the altitude range between 1300 and 1600 m (Figure 2), where attacks are not severe. This suggests that, currently, the spruce forests of the UBOR located in the high mountain and subalpine belts are free from massive bark beetle outbreaks, confirming the findings of Faccoli and Bernardinelli (2014) [7]. It also indicates that current efforts to mitigate damage should focus on submountain and mountain areas. However, based on the future climate models considered in this research, it is likely that in the coming decades, the bark beetle will find optimal climatic conditions for intense outbreaks, even in the high mountain vegetation belt, which is above 1800 m a.s.l. in the lower Camonica Valley and above 1500 m a.sl. in the upper Camonica Valley. If this occurs, it will result, within the next 60 years, in the near-total destruction/change of the current pure spruce forests in the lower Camonica Valley (where there are also few areas above 1800 m for the spruce forest to expand). Meanwhile, in the upper Camonica Valley, spruce forests will persist above 1800 m and in areas that may develop at higher altitudes, such as abandoned alpine and subalpine pastures [60].

The absence of current bark beetle infestations in the (few) spruce forests found at the lowest elevations of the UBOR (between 300 m and 600 m a.s.l.) (Figure 2) is probably due to the fact that these secondary forests, mostly small in size, are fragmented and scattered within other forest formations of the hilly belt (broadleaf forests), making them difficult for the bark beetle to locate.

The results of the floristic–vegetational and ecological analysis of the phytosociological relevés provided a series of insights into the current characteristics of forests affected by the bark beetle, suggesting the composition/type of future forest communities of the mountain belt. Except in rare cases, these are likely to be very different from those of *Calamagrostio arundinaceae-Piceetum*.

The plant communities of the forests attacked by the bark beetle were all found to be very different from those not attacked (Figure 6) because the death of the spruce trees (which constituted the dominant component of the plant community) altered a series of environmental variables in the ecosystem, favoring the growth of plant species different from those of *Vaccinio-Piceetea* and initiating a progressive secondary succession [61].

One of the ecological variables that changed most due to bark beetle attack is the availability of light in the understory, which is greater in areas affected by the pest due to the loss of leaves from dead spruces. This was confirmed by the application of the L index of Landolt et al. (2010) [48], whose values, in all sampling sites, showed post-disturbance communities to be more heliophilous than pre-disturbance ones, sometimes with very marked differences (Figure 8).

**Figure 9:**
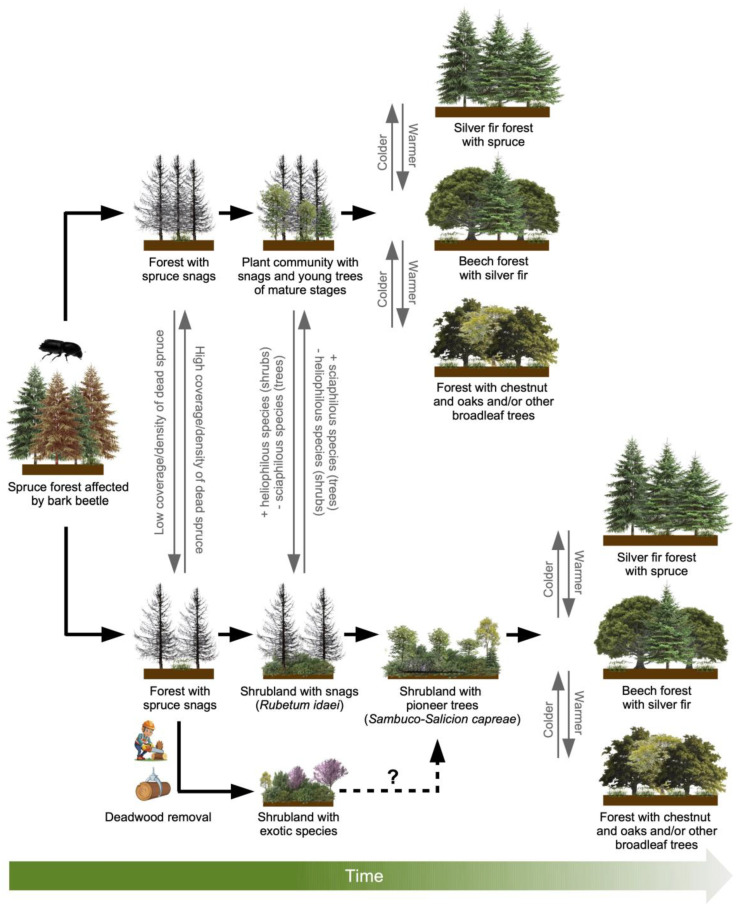
Scheme of the vegetation dynamics of submountain and mountain spruce forests affected by bark beetle in the UBOR. The black arrows with solid lines indicate the presumed dynamic relationships between the plant communities, while the black arrows with dotted lines indicate hypothetical paths that vegetation succession could follow. The grey arrows indicate the main ecological differences between the plant communities.

The different availability of light in the understory, mainly due to the coverage/density of spruce snags, appears to be one of the main factors determining the establishment of *Rubetum idaei* (cluster A1) and its variants (A3 and A4), rather than the A2 community (Figure 6b). The plant communities of clusters A1, A3, and A4 are composed of various heliophilous pioneer species of forest-clearing (*Robinietea* class), which distinguish them from A2, characterized by less heliophilous species and which, already in this early stage of secondary succession, has young trees, herbs, and shrubs of mature forest communities. A good degree of shade due to the high coverage/density of spruce snags seems to accelerate ecological succession, bypassing the *Rubetum idaei* stage and achieving the final stage (natural potential vegetation) more quickly, which is represented by broadleaved forests (chestnut, oaks, and other hardwoods) at lower elevations, beech and silver fir forests at mid elevations, and silver fir and spruce forests at higher elevations (Figure 9). The ecological role of “succession accelerator” played by communities with spruce snags deserves further study, considering it is likely that, due to bark beetle, these vegetation types will become widespread in the Southern Alps in the coming decades. Moreover, studying these ecosystems could provide important information for managing areas affected by the bark beetle and defining new nature-based solutions (NBSs)—defined as “actions inspired by, supported by or copied from nature” [62]—for forest restoration.

Based on the data collected in this research and field observations, it seems that the plant community with spruce snags could perform an ecological function similar to that of *Piceo-Sorbetum aucupariae* (*Robinietea* class), which is the stage of the acidophilus dynamic series of silver fir and spruce (*Calamagrostio arundinaceae-Piceo excelsae sigmetum*) that, in the study area, precedes the potential natural vegetation of *Piceion excelsae* [32].

Although the bark beetle-affected forests of the UBOR fall within the *Calamagrostio arundinaceae-Piceo excelsae sigmetum*—which includes the *Calamagrostion arundinaceae* (fringe) stage, followed by a shrub stage of *Sambuco-Salicion capreae* (*Rubetum idaei*, *Piceo-Sorbetum aucupariae*) and the spruce forest (potential natural vegetation) [32]—it is likely that, given the current environmental/climatic conditions, the final stage (spruce forest of *Piceion excelsae*) cannot be reached. Indeed, the plant communities of all areas attacked by the bark beetle were found to be more thermophilous (some markedly so) than those of undisturbed spruce forests (Figure 8) and often composed of young trees of mature forest communities other than spruce, such as chestnut, beech, and silver fir (clusters A2 and A4). The presence of these young trees and a complex of other species (trees, shrubs, and herbs), typical of mature forests of broadleaf trees of the *Carpino-Fagetea sylvaticae* and *Quercetea robori-petraeae* phytosociological classes (Figure 7), suggests new types of “current potential vegetation” [63] outlined in Figure 9. Specifically, it is likely that under current climatic conditions and those predicted for the coming decades, the submountain and mountain spruce forests attacked by the bark beetle in the study area could gradually (and spontaneously) be replaced by chestnut and/or oak forests in the submountain belt, beech and silver fir forests in the mountain belt, and silver fir forests with spruce in the high-mountain belt (Figure 9). The presence of indicator species of various types of mature forests (and likely different types of current potential vegetation) justifies a higher floristic–physiognomic variability between sites in the group of stands affected by bark beetles compared to unaffected forests (Figure 6a). Spruce-dominated forests are likely to be primarily located in the subalpine belt, which will expand due to the upward shift of the tree line.

This deduction is supported not only by the results of this research but also by the fact that in Europe (and other parts of the world), a significant upward shift in forest plant species and vegetational belts is being observed and modeled [14,64,65,66]. In particular, Lenoir et al. (2008) [64] detected an upward shift in species optimum elevation, averaging 29 m per decade in western Europe, and Zischg et al. (2021) [66] modeled an upward shift of vegetation belts in Swiss forests, more or less pronounced depending on the different climate change prediction models and the different topoclimatic characteristics of the territory considered. Furthermore, in the UBOR, Giupponi et al. (2023) [14], following the destruction of spruce forests by Storm Vaia, observed *Quercus petraea* (which has an ecology similar to chestnut) growing at 1300 m a.s.l., 300 m higher than the altitude at which oak/chestnut forests are mapped in the Italian vegetational series map of Blasi (2010) [32]. Indeed, changes in the altitudinal zonation of vegetation are complex and also depend on local factors other than temperature, such as soil conditions and the ability of species to outcompete the existing vegetation at higher elevations. So, studies on the topic should carefully consider local conditions when trying to predict the fate of vegetation under the rapidly changing conditions of this century.

In the coming years, it would be advisable to monitor changes in the forest communities of the UBOR to confirm/integrate the scheme in Figure 9 so that it can become a useful tool for technicians and forest managers in the study area (and the Southern Alps in general) to understand vegetation potentials and adopt correct measures and actions for sustainable management of the forest cover. Specifically, it would be beneficial to collect floristic data and better define the mature forest communities (and the stages of dynamic series) that will be present where spruce forests currently attacked by the bark beetle and/or particularly susceptible to the insect are located (Figure 4). Based on the floristic data collected in this research, it is likely that the beech forest communities that will replace spruce forests on basic soils (cluster B4) will be significantly different from those that will grow on acidic soils (e.g., replacing cluster B2). Indeed, the former are likely to be forests of *Aremonio-Fagion*, and the latter of *Luzulo-Fagion sylvaticae* [15,65]. More data and studies aimed at defining the current (and future) vegetational potentials of spruce forests attacked by the bark beetle in the Southern Alps will undoubtedly be useful, if only to update the forest type maps of Lombardy [67] and other vegetational maps of the Italian Alps, which are very useful tools for territorial management, provided they are not too outdated, given the rapidity of climate change.

Knowing the current and future vegetation potentials of areas that are (or will be) attacked by the bark beetle could suggest which species to use for the restoration of forest communities. This action should be carried out quickly (preferably using native species), at least in bark beetle-affected forests located on steep slopes. Indeed, the rapid and simultaneous death of all (or most) trees in a pure spruce forest leads, within a few years, to the decomposition of their roots, which could cause significant hydrogeological instability problems since it is well known that tree roots contribute significantly to stabilizing the soil on mountain slopes [68,69,70]. More information should be gathered on the biotechnical characteristics of the roots of herbs and shrubs that could be used for environmental restoration and soil bioengineering interventions [71].

It would also be important to conduct studies to identify bioindicators that can help more accurately determine which forests might be more susceptible to the bark beetle than species distribution models currently allow. In this sense, this research identified *Saxifraga cuneifolia* as a diagnostic species of spruce forests not attacked by the bark beetle (Table 2). This species, together with a high coverage of mosses, appears particularly associated with microthermal forests, and its absence (or low coverage) in forests not yet attacked by the pest seems to indicate high susceptibility of the spruce to the insect. *S. cuneifolia* is indeed a species of *Vaccinio-Piceetea* [16,46,47] widespread throughout the Alps [72] that is microthermal, shade-loving, and requires soil with good availability of humus/litter [48,73,74]. In the study area, it is absent (or with very low coverage values) in spruce forests not yet attacked by the bark beetle, where thermophilic broadleaf trees (chestnut and/or beech) are present (Table S2). The actual effectiveness of *S. cuneifolia* as an indicator of bark beetle-susceptible forest should be further investigated in studies that also consider other study areas in the Southern Alps, as well as analyzing the presence/absence of individual moss species in spruce forests under different stress conditions to understand if some of them could also behave similarly to *S. cuneifolia*. *Vaccinium myrtillus* also proved to be a diagnostic species of forests not attacked by the bark beetle, but the latter is not an exclusive species of *Vaccinio-Piceetea,* as it also contributes to the formation of forest communities of *Quercetea robori-petraeae* (Table 2) and is, therefore, indicative of acidophilic forest communities in general.

If *S. cuneifolia* appears to be an indicator of less stressed and less bark beetle-susceptible spruce forests, the presence of exotic species is certainly an indicator of a degraded environment, often caused by anthropogenic interventions/disturbances [75]. An example is the plant community of cluster A3, which is the result of recent cutting and removal actions of spruce snags that were dangerous for roads and houses near the forest affected by the bark beetle (sampling site 4). This community is indeed to be considered a degraded variant of *Rubetum idaei* (which developed due to the low density/coverage of spruce snags) in which various exotic species with high coverage are present—including *Senecio inaequidens* (Table S2), the only African species present in the UBOR—and rapidly spreading [27,31]. Since it is very likely that the spread of exotic species present in cluster A3 is due to the use of tools/machinery from the forestry site that carried out the cutting and removal work of the spruce snags that were contaminated with seeds of exotic plants, it would be advisable, if similar operations are to be carried out in other bark beetle-affected forests, to require forestry workers to clean their tools/machinery before moving to intervention areas, especially if these are located in protected areas or areas with few exotic species, as is the case in most mountain areas of the Alps [76].

This action, in addition to preserving the integrity of Alpine ecosystems, would also enable better utilization of their benefits/services. In fact, shrublands dominated by *Rubus idaeus* and other species of *Robinietea* class (such as those that develop in areas affected by bark beetles with low density/coverage of spruce snags) are valuable for the production of edible fruits and, even more so, for the foraging of the domestic bee (*Apis mellifera* L., Hymenoptera: Apidae) and wild pollinators, whose survival is currently threatened, among other factors, by climate change [77,78]. In the Alps, bees not only feed on the nectar of *R. idaeus* (and other nectar-producing species) but also facilitate the production of raspberry honey, a prized agri-food product with interesting phytochemical and nutritional characteristics [79]. However, the presence of *S. inaequidens* (alongside *R. idaeus*) in cluster A3 prevents the production of high-quality honey because this species contains toxic compounds (pyrrolizidine alkaloids) that contaminate honey through pollen. These toxins can enter the food chain and pose a risk to human health [80]. This is one of the reasons why *S. inaequidens* is included on the “Black List” of invasive alien species subject to monitoring and containment in the Lombardy region (Regional Law 10/2008).

From the perspective of vegetation dynamics, it is likely that the A3 community plays a role similar to that of the typical *Rubetum idaei*, but it would be prudent to monitor it over time to confirm the dynamic described in the vegetation scheme of Figure 9. Communities with *S. inaequidens*, *Buddleja davidii,* and other exotic pioneer/invasive species are relatively new to the Alps, and their role in succession is still unclear, especially considering the current and future effects of climate change.

## 5. Conclusions

This research has clarified the main environmental characteristics of the spruce forests in the UBOR that are affected by and/or susceptible to bark beetle attacks. The most impacted forests were found to be those located below 1600 m a.s.l. (in the submountain and mountain belts) and in areas with high solar radiation. Additionally, through the development of a MaxEnt model with high predictive accuracy, primarily defined by the mean temperature of the winter driest quarter, it was found that over 58% of the current spruce forests in UBOR will have a high susceptibility to intense bark beetle attacks in the next 60 years. The analysis of floristic–vegetational and synecological characteristics suggested that in areas attacked by the bark beetle, mature forest communities that are more thermophilic and significantly different (both floristically and physiognomically) from the pre-disturbance spruce forests will develop. Furthermore, based on the interpretation of the results obtained, a model of plant succession was developed that, along with the following suggestions/precautions, could be useful for land managers in order to limit damage from bark beetles, promote rapid forest regeneration, and prevent the degradation of forest ecosystems in the Southern Alps:

- Focus on measures/interventions that mitigate the spread of the bark beetle, especially in spruce forests of the submountain and mountain vegetational belts (particularly in areas with higher winter temperatures and high solar radiation), as these are the areas with the most favorable climatic conditions for the development of intense pest outbreaks;
- Promote the conversion of pure spruce forests into mixed forests, prioritizing native species and considering both their ecology and economic value;
- Avoid the spread/planting of spruce outside its natural/ecological range, where it could experience more stress and be more vulnerable to bark beetle attacks and/or other pests/diseases;
- Encourage, where possible, the spread of spruce in the subalpine belt, where it is unlikely that favorable climatic conditions for intense bark beetle attacks will occur in the coming decades;
- Promote rapid actions/interventions to facilitate tree growth in forests affected by the bark beetle on steeper slopes to prevent hydrogeological instability;
- Avoid the removal of spruce snags (in forests affected by the bark beetle) if the goal is to accelerate vegetation succession and achieve mature forest communities more quickly (which may differ significantly from pre-disturbance communities);
- In cases of spruce snag removal (for protective, productive, and/or ecological–conservation purposes) or other anthropogenic interventions, ensure that measures are adopted to contain exotic species, such as cleaning tools/machinery before their transport/use in the intervention area.

These tools/suggestions represent an initial contribution to supporting a more informed and sustainable management of areas affected by and/or susceptible to the bark beetle in UBOR and areas with similar environmental conditions. It is to be hoped they will soon be integrated with the results of other research, given the speed at which the bark beetle is spreading in the Southern Alps and the magnitude of the effects it is causing to their forest ecosystems and landscapes.

## Figures and Tables

**Table 1:** Bioclimatic variables used to model the distribution of *I. typographus* epidemics in the UBOR. In the description of bioclimatic variables, the term ‘quarter’ refers to a period of three consecutive months within a year.

Code/Unit	Bioclimatic Variable
BIO1 (°C)	Annual Mean Temperature
BIO2 (°C)	Mean Diurnal Range (Mean of monthly, max-min T)
BIO3 (-)	Isothermality (BIO2/BIO7 × 100)
BIO4 (°C)	Temperature Seasonality (standard deviation × 100)
BIO5 (°C)	Max Temperature of Warmest Month
BIO6 (°C)	Min Temperature of Coldest Month
BIO7 (°C)	Temperature Annual Range (BIO5-BIO6)
BIO8 (°C)	Mean Temperature of Wettest Quarter
BIO9 (°C)	Mean Temperature of Driest Quarter
BIO10 (°C)	Mean Temperature of Warmest Quarter
BIO11 (°C)	Mean Temperature of Coldest Quarter
BIO12 (mm)	Annual Precipitation
BIO13 (mm)	Precipitation of Wettest Month
BIO14 (mm)	Precipitation of Driest Month
BIO15 (-)	Precipitation Seasonality (Coefficient of Variation)
BIO16 (mm)	Precipitation of Wettest Quarter
BIO17 (mm)	Precipitation of Driest Quarter
BIO18 (mm)	Precipitation of Warmest Quarter
BIO19 (mm)	Precipitation of Coldest Quarter

**Table 2:** Diagnostic species of cluster A (forest affected by bark beetle) and cluster B (forest not affected by bark beetle). Phytosociological class code, Pearson’s phi coefficient (*Φ*), and *p*-value of each diagnostic species are reported. The codes of phytosociological class are the same used by Mucina et al. (2016) [16]: PIC, *Vaccinio-Piceetea*; ROB, *Robinietea*; FAG, *Carpino-Fagetea sylvaticae*; EPI, *Epilobietea angustifolii*; POP, *Alno glutinosae-Populetea albae*; RHA, *Crataego-Prunetea*; PAR, *Papaveretea rhoeadis*; QUE, *Quercetea robori-petraeae*; ULI, *Calluno-Ulicetea*; NAR, *Nardetea strictae*. Key: *, significant (*p* < 0.05); **, significant (*p* < 0.01).

Cluster	Diagnostic Species	Phytosociological Class Code	*Φ*	*p*-Value	
A	*Picea abies* (dead)	PIC	0.951	0.005	**
	*Rubus idaeus*	ROB	0.470	0.005	**
	*Mycelis muralis*	FAG, EPI	0.406	0.005	**
	*Fragaria vesca*	EPI	0.295	0.005	**
	*Solanum dulcamara*	POP	0.286	0.040	*
	*Sambucus racemosa*	ROB	0.285	0.015	*
	*Rubus ulmifolius*	RHA	0.270	0.005	**
	*Betula pendula*	ROB	0.266	0.005	**
	*Geranium robertianum*	FAG, EPI	0.260	0.015	*
	*Galium aparine*	POP, EPI	0.234	0.015	*
	*Taraxacum officinale* (group)	-	0.226	0.010	**
	*Sambucus nigra*	ROB, POP	0.205	0.005	**
	*Galeopsis tetrahit*	PAR, EPI	0.186	0.005	**
	*Buddleja davidii*	ROB	0.167	0.005	**
	*Salix caprea*	ROB	0.144	0.005	**
	*Urtica dioica*	ROB, POP, EPI	0.142	0.005	**
B	*Picea abies* (alive)	PIC	0.990	0.005	**
	Mosses	-	0.309	0.030	*
	*Vaccinium myrtillus*	PIC, QUE, ULI, NAR	0.280	0.030	*
	*Saxifraga cuneifolia*	PIC	0.228	0.040	*

## Data Availability

The raw data supporting the conclusions of this manuscript will be made available by the corresponding author, without undue reservation, to any qualified researcher.

## Supplementary Materials

The following supporting information can be downloaded at: https://www.mdpi.com/article/10.3390/plants14111681/s1, Table S1: Percentage contribution and permutation importance of the predictor variables to the MaxEnt model; Table S2: Phytosociological table of relevés. Cover indices refer to the [39] abundance/dominance scale: r, rare; +, <1%; 1, 1–5%; 2, 6–25%; 3, 26–50%; 4, 51–75%; 5, 76–100%; Table S3: Average values of ecological indices for vegetation types (forests affected and unaffected by bark beetles) across each sampling area. Key: T—temperature, K—continentality, L—light intensity, F—soil moisture, R—substrate reaction, N—nutrients, H—humus, D—soil aeration; *, significant (*p* < 0.05); **, significant (*p* < 0.01).
